# Clinical and Demographic Predictors of Health-Related Quality of Life After Orthopedic Surgery With Implant Placement

**DOI:** 10.7759/cureus.21348

**Published:** 2022-01-17

**Authors:** Georgia Keramari, Ioannis Moisoglou, Evangelia Meimeti, Petros Galanis, Evangelos C Fradelos, Ioanna V Papathanasiou

**Affiliations:** 1 Operating Room, General Hospital of Thessaloniki “George Papanikolaou", Thessaloniki, GRC; 2 Pulmonary Clinic, General Hospital of Lamia, Lamia, GRC; 3 Training Coordination: Public Health/Community Nursing in Central and Western Macedonia, 3rd Regional Health Authority of Macedonia, Thessaloniki, GRC; 4 Center for Health Services Management and Evaluation, Faculty of Nursing, National and Kapodistrian University of Athens, Athens, GRC; 5 Nursing, University of Thessaly, Larissa, GRC; 6 Community Nursing Lab, Faculty of Nursing, University of Thessaly, Larissa, GRC

**Keywords:** orthopedic surgery, surgery, sf-36, orthopedic, health-related quality of life

## Abstract

Background: Orthopedic surgeries can rehabilitate injuries and at the same time improve the patients’ quality of life. The study aimed to assess patients’ health-related quality of life (HRQOL) six months after an orthopedic surgery with implant placement.

Materials and methods: A cross-sectional study with the use of a structured questionnaire among 103 patients was conducted. The 36-Item Short Form Survey (SF-36) questionnaire was used to evaluate patients’ quality of life.

Results:According to the findings of the multivariate linear regression analysis, low age, marital status (married in comparison to unmarried/ divorcees/widows), reduced intensity of the pain, and low educational attainment were associated with a better quality of life. Furthermore, the patients who were living with another person and the patients who underwent surgery on a part of the body other than the hip presented better quality of life. The results of the multivariate analysis explained 33%-67% of the variance of the SF-36 HRQOL.

Conclusion: Measuring quality of life is a valuable asset that helps to reveal the frail patient groups, in which health professionals will prioritize their care and the state in turn will design primary care services to meet their needs after discharge from the hospital.

## Introduction

The health-related quality of life (HRQOL) measures the effect of a disease and its symptoms in patients’ quality of life (QOL) [1]. The patient can be often found trapped in a vicious cycle where the disease’s symptoms (causal indicators) can affect the patient's QOL. Through this negative effect, new problems arise (effect indicators) and turn into causal indicators that have a negative effect on the QOL [2]. Apart from the disease itself and its symptoms, there is also a significant number of individual and environmental characteristics that can have an impact on health outcomes and they should be examined during the studies on HRQOL [3].

According to the World Health Organization (WHO), musculoskeletal diseases are the first cause of disability worldwide. It is estimated that about 20%-33% of the total population suffers from a painful musculoskeletal condition [4,5]. The most common and disabling musculoskeletal condition is osteoarthritis, which often leads to orthopedic surgeries, back and neck pain, fractures associated with bone fragility, injuries, and systemic inflammatory conditions such as rheumatoid arthritis, whose rehabilitation and treatment require large amounts of money [5]. The aforementioned conditions affect the patients’ QOL as well: they limit their daily activities, reduce their ability to work and participate in social roles, and have an impact on their mental wellbeing.

Literature review

Orthopedic surgeries can restore some of the damage and improve a patient’s QOL, thus reversing the limitations and the pain the patient experiences. A prospective study in patients who underwent a hip and knee replacement surgery initially revealed a significant improvement in physical function and pain six months after the surgery, while at the end of the study (seven years follow-up) an improvement was reported in role physical and role emotional [6]. The fact that QOL remains high for years after the surgery is a very important finding and emphasizes the importance of such surgical procedures [7]. Patients who were prospectively observed for 24 months after a high tibial osteotomy showed a significant improvement in HRQOL and a decrease in pain during a six-month follow-up. Functional outcomes increased significantly in a 12-month follow-up and, at the final follow-up, 90% of the patients returned to their previous occupation without limitations [8].

However, several studies have shown that QOL does not always improve in patients who have undergone orthopedic surgery. Among the main factors that can have a negative effect on patients’ QOL are sex, age, pain, and the body mass index (BMI). Data analysis on a large sample in the Netherlands showed that females and people with a higher BMI experienced some pain post-surgically. Advanced age and high BMI were related to lower QOL post-surgically, lower functionality, and more pain [9]. BMI is also associated with the average length of patients' stay at the hospital after orthopedic surgery, as obese patients required one more day of hospitalization and they presented a higher probability of readmission within 30 days after their discharge [10]. Patients with moderate to severe persistent pain have reported more limitations on their daily activities. Nevertheless, these limitations have not affected their QOL. On the contrary, severe pain and BMI were found to affect patients’ daily functionality six months post-operatively [11].

The assessment of QOL and the investigation of the factors that can affect it have multiple benefits for the patient, the physician, and the health system. Studies revealed the factors and the vulnerable patient groups that require the attention of health-care professionals so that they will be able to provide integrated pre-operative and post-operative care to cure the patients and improve their QOL. Regarding the health system, such studies can help the policymakers in decision-making about the design of health services that are suitable to meet the care needs of these patients in the community.

The study aimed to assess patients’ HRQOL six months after an orthopedic surgery with implant placement.

## Materials and methods

Study design

A cross-sectional study using a structured questionnaire was conducted. The convenience sample consisted of patients who had undergone orthopedic surgery with implant placement in a Greek public general hospital. The questionnaire was completed after completion by patients’ follow-up visit at the outpatients’ orthopedic department, which is six months after their surgery. The study was conducted through phone interviews and lasted from March 20th to April 30th, 2020. A total of 103 patients were chosen to participate (convenience sampling). All patients agreed to participate (response rate 100%). The inclusion criteria were the following: 18 or more years of age, ability to communicate in the Greek language, emergency, and scheduled surgeries. The operations were performed at the study hospital (Greek public general hospital). The patients, after the end of six months from the operation, were examined in the orthopedic outpatient clinic of the same hospital, in order to assess their post-operative condition. 

Ethics 

The study protocol was approved by the Ethical Committee of the participated hospital. The patients were approached by a member of the research team and asked if they wanted to participate in the study. Participants were informed about ethics issues before completing the questionnaires. In particular, they were informed that their participation is voluntary, anonymous, the collected data will be used exclusively for the purposes of the survey, and that they can leave the completion of the questionnaires at any time they wish. This study was conducted in compliance with the ethical standards of the responsible institution on human subjects as well as with the Helsinki Declaration.

Study measures

The 36-Item Short Form Survey (SF-36) [12], which assesses the QOL through 36 questions, was used to measure patients’ QOL. The questionnaire had been translated and validated in Greek [13]. The questionnaire consists of eight sub-scales, which comprise two wider concise areas: physical and mental health. Cronbach’s alpha of the Greek validated version of the instrument ranged from 0.79 (Social Functioning) to 0.95 (Role Physical), exceeding, in all cases, the 0.70 standard for group-level comparisons. A series of demographic and clinical characteristics such as age, gender, and marital status were also recorded. 

Statistical analysis

Categorical variables are expressed as mean (n) and relative (%) frequency, while quantitative variables are expressed as mean value, standard deviation, median, minimum value, and maximum value. The Kolmogorov-Smirnov test and graphs (histograms and normal Q-Q plots) were used to assess the normality of the distribution of the quantitative variables. The independent variables were the demographic and clinical characteristics, while the dependent variables were the scores on the SF-36.

The t-test (students’ t-test) was used to assess the association between a quantitative variable and a binary variable, whereas the analysis of variance was used to assess the association between a quantitative variable and a qualitative variable with >2 categories. Pearson’s correlation coefficient was used to assess the association between two quantitative variables. Spearman’s correlation coefficient was used to assess the association between a quantitative variable that is normally distributed and a quantitative variable that is not normally distributed.

In the cases where >2 independent variables were statistically significant (p < 0.2) in bivariate analysis, a multivariate linear regression analysis with the scores as the dependent variables was applied. In this case, the backward stepwise linear regression was used. Regarding the multiple linear regression the coefficients’ beta, the respective 95% confidence intervals, and p values are presented. In the case where the scores on the SF-36 did not follow a normal distribution, logarithms were used. The results are presented on a natural scale and not in the logarithmic scale to be simplified and comprehensible.

The two-sided level of statistical significance was set at 0.05. Data analysis was performed using the IBM SPSS 21.0 (Statistical Package for Social Sciences; IBM Corp, Armonk, NY).

## Results

The study population included 103 patients whose demographic characteristics are presented in Table 1. The patients’ mean age was 67.2 years, while the majority consisted of females (63.7%), married (59.2%), with children (80.6%), primary school graduates (48%), pensioners (64.1%), insured (93.2%), and lived with their spouse (55.9%).

**Table 1:** Demographic characteristics of the participants (n = 103). *Mean (standard deviation).

Characteristics	Ν (%)
Sex	
Male	37 (36.3)
Female	65 (63.7)
Age	67.2 (14.5)*
Marital Status	
Unmarried	12 (11.7)
Married	61 (59.2)
Divorcees	3 (2.9)
Widows	27 (26.2)
Number of Children	
0	20 (19.4)
1	21 (20.4)
2	48 (46.6)
3	12 (11.7)
4	2 (1.9)
Educational Attainment	
Uneducated	7 (6.9)
Primary School	49 (48)
Junior High School/High School	32 (31.4)
University	14 (13.7)
Employment Status	
Employed	25 (24.3)
Unemployed	12 (11.7)
Pensioners	66 (64.1)
Cohabitation	
Alone	34 (33.3)
Spouse	57 (55.9)
Children	9 (8.8)
Care Home	1 (1)
Parents	1 (1)
Insurance	
Yes	96 (93.2)
No	7 (6.8)

The patients’ clinical characteristics are presented in Table 2. The majority of them underwent hip surgery (40.8%); knee and hip surgeries were arthroplasty (joint replacement cases) and all other cases were fracture repair cases, and did physical therapy (80.6%), the average of which was 13. A percentage of 57.3% was experiencing pain and the average pain rating was 4.3 (on a scale from 0 to 10). A percentage of 24% were smokers and 43.7% had comorbidity. The most frequent comorbidities were hypertension, diabetes, coronary disease, cancer, and renal failure. Regarding the comorbidity score according to the Charlson Index [14], 56.3% scored 0, 32% scored 1, 9.7% scored 2, 1% scored 3, and 1% scored 4.

**Table 2:** Patients’ clinical characteristics (n = 103). *Mean (standard deviation).

Characteristic	Ν (%)
Type of Surgery	
Lower Leg	12 (11.7)
Tibia/Fibula	8 (7.8)
Knee	25 (24.3)
Hip	42 (40.8)
Arm/Shoulder	16 (15.5)
Physical Therapy (PT)	
Yes	83 (80.6)
No	20 (19.4)
Number of PT	13 (10.2)*
Second Surgery After Orthopedic	
Yes	5 (5)
No	96 (95)
Pain	
Yes	59 (57.3)
No	44 (42.7)
Pain Intensity	4.3 (1.7)*
Smoker	
Yes	24 (24)
No	76 (76)
Comorbidity	
Yes	45 (43.7)
No	58 (56.3)
Hypertension	34 (33)
Diabetes	21 (20.4)
Coronary Disease	8 (7.8)
Cancer	4 (3.9)
Renal Failure	4 (3.9)
Charlson Comorbidity Score Index	
0	58 (56.3)
1	33 (32)
2	10 (9.7)
3	1 (1)
4	1 (1)

The internal consistency coefficients (Cronbach’s alpha) for the eight subscales of SF-36 are presented in Table 3. The minimum acceptable value for Cronbach's alpha internal consistency coefficient was >0.7. In all scales, alpha was >0.70, which shows a very good internal consistency.

**Table 3:** Cronbach's alpha for the scales and overall SF-36. SF-36, 36-Item Short Form Survey.

Scale	Cronbach's Alpha
Physical Function	0.92
Role Physical	0.70
Bodily Pain	0.74
General Health	0.83
Vitality	0.82
Social Functioning	0.72
Role Emotional	0.76
Mental Health	0.85
Overall	0.91

The descriptive results for the scores in the SF-36 are presented in Table 4. Both the physical health component summary and the mental health component summary were lower than 50, which shows an inferior QOL in comparison to the general population. Moreover, the mental health component summary scored higher than the physical health component summary, which shows that the patients’ mental health was better than their physical health.

**Table 4:** Descriptive statistics for the scores on the SF-36 subscales. SF-36, 36-Item Short Form Survey.

Scale	Mean	Standard Deviation	Median	Minimum Value	Maximum Value
Physical Function	45.8	32.1	45	0	100
Role Physical	42	46.3	0	0	100
Bodily Pain	67.9	27.9	74	0	100
General Health	55.8	28.3	60	0	100
Vitality	56.7	28.3	60	0	100
Social Functioning	71.2	31.7	75	0	100
Role Emotional	48.9	45.9	33.3	0	100
Mental Health	65.1	26.9	72	4	100
General Well-being	38.8	11.5	40	17	58.6
General Mental Health	46.9	13.1	48.9	15	65

Bivariate analysis between the demographic and clinical characteristics using the scores of the eight subscales of the questionnaire was conducted. Following the bivariate analysis, multivariate linear regression was applied to the independent variables that presented an association of 0.20 (p < 0.20). The results are presented in Table 5. According to the findings of the multivariate linear regression analysis, low age, marital status (married in comparison to unmarried/divorcees/widows), reduced intensity of the pain, and low educational attainment were associated with a better QOL. Also, the patients who lived with another person and the patients who underwent surgery on a part of the body other than the hip presented a better QOL. The results of the multivariate analysis explained 33%-67% of the variance of the SF-36 HRQOL.

**Table 5:** Multivariate linear regression analysis with the demographic and clinical characteristics as the independent variables and the scores of the subscales as dependent variables.

Dependent and Independent Variables	Coefficient Beta	95% Confidence interval for Beta	p-Value	Adjusted R^2^
General Well-being				66%
Age	-0.4	-0.6 to -0.2	0.001	
Married vs Unmarried/Divorcees/Widows	5.2	1.6 to 8.8	0.006	
Pain intensity	-1.9	-3 to -0.9	0.001	
Other Operated Body Members vs The Hip	5.4	1.6 to 9.2	0.006	
General Mental Health				45%
Age	0.3	-0.5 to -0.04	0.021	
Married vs Unmarried/Divorcees/Widows	7.5	2.1 to 12.9	0.008	
Other Operated Body Members vs The Hip	9.5	3.9 to 15.3	0.001	
Physical Function				62%
Age	0.7	1.1 to 0.3	0.001	
Married vs Unmarried/Divorcees/Widows	14.5	3.9 to 25.1	0.008	
Pain intensity	3.6	6.7 to 0.6	0.021	
Other Operated Body Members vs The Hip	21.1	10.1 to 32.1	0.001	
Role Physical				33%
Age	-1.4	-2.5 to -0.3	0.016	
Married vs Unmarried/Divorcees/Widows	29	9.5 to 48.5	0.004	
Educational Attainment	-20	-36 to -3	0.023	
Bodily Pain				56%
Married vs Unmarried/Divorcees/Widows	9.5	0.7 to 18.3	0.040	
Pain intensity	-5.8	-8.3 to -3.2	0.001	
Other Operated Body Members vs The Hip	17.6	9.1 to 26.1	0.001	
General Health				67%
Age	0.9	-1.3 to -0.6	0.001	
Married-Unmarried/Divorcees/Widows	13	3.9 to 22.1	0.006	
Pain intensity	-3.9	-6.5 to -1.2	0.005	
Other Operated Body Members vs The Hip	11.9	2.4 to 21.3	0.015	
Vitality				55%
Age	-1.1	-1.7 to -0.5	0.001	
Married vs Unmarried/Divorcees/Widows	13.5	2.8 to 24.2	0.015	
Other operated body members vs the hip	12.9	1.5 to 24.1	0.027	
Social Functioning				56%
Age	-2.1	-3.1 to -1	0.001	
Married vs Unmarried/Divorcees/Widows	23.8	3.8 to 43.7	0.021	
Other Operated Body Members vs The Hip	25	11.5 to 38.7	0.001	
Role Emotional				34%
Cohabitation vs Living Alone	34.2	13.6 to 54.8	0.002	
Other Operated Body Members vs The Hip	33.9	13.6 to 54.1	0.001	
Mental Health				42%
Married vs Unmarried/Divorcees/Widows	16.5	5 to 27.9	0.006	
Other Operated Body Members vs The Hip	19.3	7.2 to 31.3	0.002	

## Discussion

The present study investigated patients’ QOL six months after an orthopedic surgery with implant placement. According to the results of the study, both the physical health component summary and the mental health component summary were lower than 50, which shows an inferior QOL in comparison to the general population. These findings can be interpreted by the fact that often patients after orthopedic surgery need time to achieve a restoration of their QOL, and often a longer period of six months is required, which can reach years later [6,7]. Also, as most of the participants report the existence of pain (with an average pain intensity value of 4.3), are elderly, and suffer from comorbidities, all of these may create significant limitations in their daily lives with an impact on their QOL. Regarding the participants’ demographic characteristics, the young, the married, and those who lived with their partner were found to have a better QOL and presented better results in all the scales of the questionnaire. A person’s ability to perform his daily family and social activities demands good physical health and good physical function, to a great extent. The support and the help that other family members can provide can contribute to a more effective fulfillment of these efforts. Patients who live alone present more functional limitations after orthopedic surgery [15]. Also, the post-operative physical function, social function, and vitality are affected by the patients’ age; older people experience greater impairment of their QOL associated with the above scales [16]. The benefits associated with marital status do not affect only the orthopedic patients. They can have a positive effect on the survival of oncological patients as well [17]. According to a recent study in the population of Sweden, married people have up to 2 times lower probability of mortality of COVID-19 in comparison to unmarried people or people who live alone [18]. The implementation of interdisciplinary exercise schedules in the community to improve the strength of the elderly has proven effective, as it can improve the patients’ pain level, physical function, role physical, vitality, social networking, and mental health [19-21], supported by the regions or municipalities, have also proven beneficial for the elderly or lonesome people of the community. Such programs offer various primary care services such as personal help and adult day-services, which help people with disabilities live in the community [22]. Many municipalities in Greece implement a similar program called “In-House Help”. This primary care program provides help and care to the elderly and chronic patients who participate in order to cover their physical, mental, and sentimental needs. Several studies have proven the program’s efficiency in covering the aforementioned needs of the participants [23,24].

According to the clinical characteristics in the present study, patients who underwent surgery on the hip and experienced post-operative pain of high intensity presented an inferior QOL. High-intensity post-operative pain is common in orthopedic surgeries and can reduce patients’ functionality and health level, affect their QOL, and can even result in mortality [25,26]. The present study’s findings partially coincide with the respective findings of a study on total hip replacements, where a total of 104 patients participated six months after their surgery. A percentage between 26% and 58% admitted that they experienced persistent pain on the hip [11]. The patients who experienced moderate to severe pain also reported more limitations on their daily activities. However, those limitations were not found to affect the patients’ QOL.

A hip replacement surgery can cause severe mobility difficulties, which explains why patients who underwent surgical procedures other than hip replacement reported a better QOL in the present study. Regardless of the good function of other body members, mobility limitations also impede any activity. The limitations of hip surgery are more determinant in comparison to the limitations of surgical procedures in other parts of the lower extremities [27]. The improvement of the patients’ functionality, hence the improvement of their QOL after a hip replacement, can include threefold interventions. The first one refers to the reduction of waiting for surgery; waiting affects QOL, thus affecting the post-surgical outcome [28]. The other two interventions refer to the interdisciplinary cooperation for the patient’s optimum care, which does not only include the orthopedics specialist. The cooperation of health specialists (psychologists, physical therapists, nutritionists), which should begin when the patient is admitted to the hospital and continue even after the patient has returned home, has been found to have beneficial results [29]. The benefits of the interdisciplinary interventions are important for the hospitals and the health-care system as well, as they can help reduce the average hospitalization period and the cost of care [30].

Limitations 

The current study contains a series of limitations that need to be assessed when generalizing the results. The study was conducted in only one hospital, with the participation of a relatively small sample of patients. Also, as the questionnaires were answered through phone interviews, it was impossible to estimate the patients’ BMI, which according to the literature can affect the patients’ QOL. Also, the comorbidity score has been estimated using the patients’ reports on chronic illnesses, and not their medical files. As a result, relevant information might have been omitted.

## Conclusions

The present study on patients who have undergone orthopedic surgery with implant placement has revealed a series of demographic and clinical data, which have revealed the frail patient groups and the areas where health-care professionals and the state should focus. In addition, future studies should have long-term evaluation points (follow-up 1-3 years) in order to reliably capture the value of interventions in improving the QOL of the patients, as after a period of six months, there is likely to be a small improvement in QOL. It is important to measure QOL to better examine these areas, which have a multidimensional role and can affect not only the QOL but the patients’ mortality as well. The benefits from the improvement of the QOL can expand to the health-care systems, thus reducing the required resources for patients’ care.
